# Impact of *Saccharomyces cerevisiae* on the intestinal microbiota of dogs with antibiotic-induced dysbiosis

**DOI:** 10.3389/fvets.2025.1462287

**Published:** 2025-02-06

**Authors:** Sara Arghavani, Younes Chorfi, Mariela Segura, Achraf Adib Lesaux, Marcio C. Costa

**Affiliations:** ^1^Department of Veterinary Biomedical Sciences, Faculté de médecine vétérinaire, Université de Montréal, Saint-Hyacinthe, QC, Canada; ^2^Department of Pathology and Microbiology, Faculté de médecine vétérinaire, Université de Montréal, Saint-Hyacinthe, QC, Canada; ^3^Phileo by Lesaffre, Marcq-en-Barœul, France

**Keywords:** microbiota manipulation, yeast probiotics, intestinal dysbiosis, gut microbiota, microbiome, antibiotics

## Abstract

**Introduction:**

The gut microbiota plays an important role in the health of dogs, but treatment with antibiotics causes marked dysbiosis. The objectives of this study were to evaluate the impact of yeast probiotic *Saccharomyces cerevisiae* supplementation on the fecal microbiota of dogs and its potential to prevent dysbiosis induced by antibiotics.

**Methods:**

Twenty healthy adult dogs were divided into a control and a yeast probiotic group receiving 1g/kg of *S. cerevisiae* (Actisaf^®^, Phileo by Lesaffre, Marcq-en-Barœul, France) daily from D0 to D31. Both groups were given oral metronidazole from D11 to D17. Fecal swabs were collected on D0, 3, 11, 17, 20, 24, and 31 for microbiota analysis and blood on D0 and D24 for measurements of cytokines and cortisol.

**Results and discussion:**

At D0, two distinct microbiota profiles comprised of dogs from both groups, control and probiotic, were identified. One profile had higher abundances of species related to stress and inflammation, and the other comprised species associated with good intestinal health. After three days of supplementation with yeast probiotic *S. cerevisiae*, all five dogs from the probiotic group having a stress-related microbiota (membership) shifted to a healthy microbiota. Metronidazole markedly changed the microbiota of both groups (*p* <0.001). Still, treated dogs had significantly different microbiota on D17 (end of antibiotics treatment). The dysbiosis was resolved in both groups by D24. TNF-*α* remarkably decreased from D0 to D24 (*p* = 0.002) in the probiotic group, which also had lower levels than controls on D24 (*p* = 0.040). There were no significant differences in the other measured cytokines. It was concluded that the use of yeast probiotic *S. cerevisiae* positively shifted the microbiota composition of healthy adult dogs carrying an abnormal microbial profile and that it has the potential to attenuate the dysbiosis caused by oral metronidazole.

## 1 Introduction

The gastrointestinal tract (GIT) is a complex ecosystem that contains various living microorganisms, including viruses, archaea, fungi, parasites, and bacteria. These microorganisms are collectively known as microbiota (1). The GIT microbiota plays a vital role in maintaining the host’s health by modulating the immune system, protecting from pathogens, improving intestinal barrier function, and providing essential metabolites (2, 3). It also has a critical role in the metabolism of short-chain fatty acids (SCFAs), bile acids (BA), and indole (4). The importance of the microbiota on the host’s health has been better understood after the development of DNA sequencing technologies (5).

Dysbiosis is characterized by an imbalance or a change in the standard microbiota composition. Most dogs with gastrointestinal diseases, such as diarrhea and chronic inflammatory diseases, have concurrent dysbiosis (6, 7) associated with low bacterial diversity, deficient production of metabolites, and an increased abundance of pathogenic or pathobiont species (8). Therefore, restoring normal microbiota composition is important to aid in treating diseases (9). The most used methods to correct dysbiosis are probiotics, prebiotics, fecal microbiota transplantation (FMT), and dietary modulation.

Antibiotics are used in treating gastrointestinal diseases in dogs, but also in treating other infections (e.g., ear, skin, etc.) and have been heavily overused in veterinary medicine (10). Antibiotics are not selective for pathogenic bacteria, affecting beneficial species in the GIT. The use of antibiotics causes compositional changes and a decrease in diversity, a feature of a healthy microbiota (11). However, even though the clinical signs of diarrhea are reduced after the administration of antibiotics, the dysbiosis worsens, and some dogs remain in that abnormal state for an extended period (12–14). In addition to a decreased diversity, compositional changes observed in dogs treated with antibiotics include increased abundance of *Escherichia coli* and decreased beneficial species (e.g., *Clostridium hiranonis* and *Fusobacterium* spp.) (15, 16). Therefore, probiotics have been suggested as an alternative to treating dogs with acute diarrhea (17).

Probiotics are live microorganisms that cause beneficial effects on health when administered in adequate amounts. They have been proposed to restore the microbiota of individuals with dysbiosis (18) and to aid with the prevention of diarrhea in dogs (19). The mechanisms of action of probiotics depend on their strain, some acting on immune modulation, inhibition of pathogens, or improving the intestinal barrier (20). The major bacterial strains used as probiotics in dogs are *Lactobacillus* spp. and *Bifidobacterium* spp. (21, 22). However, the viability of organisms present in commercial veterinary products and the presence of antimicrobial resistance genes have been topics of concern (23, 24). Yeasts are mainly used as food supplementation in dogs, but some species, such as *Saccharomyces* spp., have been used as probiotics (25, 26). The cell wall of *S. cerevisiae* is structured by two fractions: one is the bond of beta-1,3/1,6-glucans and chitin, and the other comprises mannan oligosaccharides (27). Mannan oligosaccharide can act as a prebiotic to increase the population of *Lactobacilli* (28) and *Bifidobacteria* (29), benefiting the host by preserving the integrity of the absorption surface of the GIT (30) and producing SCFA in dogs (31). In rat models, it was also observed that beta-glucan can stimulate the growth of *Lactobacilli* populations (32) and that mannan oligosaccharides increase the number of white blood cells and improve the host’s immune response against pathogens (33). The recommended doses for dogs can vary from 250 million to 2 billion CFUs depending on the formulation of the product and the purpose of the use (e.g., treatment of diarrhea or enhancement of immunity).

This study hypothesized that using yeast probiotic *S. cerevisiae* would change the fecal microbiota composition of healthy dogs and would be associated with a more resilient microbiota. The study aimed to evaluate the impact of yeast probiotic *Saccharomyces cerevisiae* supplementation on the fecal microbiota of dogs and its potential to prevent dysbiosis induced by antibiotics.

## 2 Materials and methods

### 2.1 Study design

Experimental procedures were performed following the Canadian Council for Animal Care guidelines and were approved by the Animal Care Committee of the Université de Montréal (#21Rech2133).

Twenty healthy adult female intact beagle dogs from a teaching colony were selected for this study. The mean weight of the dogs was 9.6 kg, and the average age was 3.5 years. All dogs were fed a commercial diet (Breed Health Nutrition Beagle Adult Dry Dog Food, Royal Canin) for over 6 months to fulfill their maintenance energy requirements. All dogs had body scores of 4 or 5 out of 9. Dogs from the same pen were released together every morning into a walking area for approximately 1 h. The study took place during the summer. None of the dogs had a history of gastrointestinal disease, nor had they received antimicrobials or other medications during the 3 months before the study. The dogs were housed in the same room in four different stalls (two on each side of the room) with five dogs each.

Dogs were divided into two groups: a probiotic group, receiving the yeast (Actisaf^®^, Phileo by Lesaffre, Marcq-en-Barœul, France) from day D0 to D31 (1 g/kg—2.9 × 10^8^ CFU/g per day, orally), and a control group not receiving any supplementation. Each group was represented by one stall on each side of the room (control-left, control-right, probiotic-left, and probiotic-right). All the dogs were treated with oral metronidazole (15 mg/kg every 12 h orally) for 5 days (from D11 to D17). Throughout the study, all dogs remained supervised by the research staff and the veterinarian responsible for the laboratory.

Fecal samples were collected from the dogs using rectal swabs (BD ESwab^™^ collection and transport system) on D0 (baseline), D3, D11 (before antibiotic treatment), D17 (last day of antibiotic treatment), D20, D24, and D31. The swabs were stored at −80°C until further analysis. Blood samples were collected on D0 and D24 (5–10 mL collected from the jugular vein) using a 10 mL syringe and transferred to a collection tube with no additives. The serum was recovered from the tubes after 4 h at room temperature and stored at −80°C until analysis.

### 2.2 Microbiota analysis

Total DNA was extracted from rectal swabs using a commercial kit (DNeasy PowerSoil Kit, QIAGEN) following the manufacturer’s instructions. Amplicons were obtained after PCR amplification of the V4 region of the 16S rRNA gene using the primers 515F (GTGCCAGCMGCCGCGGTAA) and 806R (GGACTACHVGGGTWTCTAAT). Sequencing was performed at the Genome Quebec McGill Innovation Centre using an Illumina MiSeq platform for 250 cycles from each end, aiming to overlap the reads fully.

Bioinformatic analysis was performed using the software Mothur (34) following the previously described Standard Operating Procedure (35). Contig assembly was conducted from the original fastq files, excluding sequences longer than 300 bp, containing base pair ambiguities and having polymers longer than 8 bp. The sequences were aligned using the SILVA 16S rRNA reference database and clustered at 97% similarity before chimeras were removed and classified using the Ribosomal Databank Project. Sequences classified as the same genus were clustered (Phylotypes) for further analyses (36).

The Chao and the Shannon index were used to characterize the alpha diversity. Beta diversity (comparison of taxonomic composition between samples) was assessed by the Jaccard index to evaluate community membership (that considers only the presence or absence of each bacterial taxon) and the Bray–Curtis index to evaluate community structure (that also considers how often each taxon appeared in the analysis). A two-dimensional principal coordinate analysis (PCoA) plot was generated to visualize the similarity between samples. The most abundant bacteria (>1%) were visualized by generating bar charts representing the relative abundance of the main phyla and genera found in each sample.

### 2.3 Cytokine’s analysis

The cytokines levels in the serum were analyzed using a ProcartaPlex^™^ Human, NHP, and Canine Mix & Match Panels kit in a Luminex xMAP (multi-analyte profiling) technology to enable the detection and quantitation of seven cytokines: interferon (IFN)-γ, interleukin (IL)-2, IL-6, IL-8, IL-10, IL-12, and tumor necrosis factor (TNF)-α. Serum samples were diluted before assay, and the procedure was performed according to the manufacturer’s instructions. The serum matrix solution provided in the kit was used in the sample and standard and control wells as a background. The method was validated with assay controls consisting of recombinant canine cytokines and the intra-assay precision. Luminex technology uses differential dye to capture beads for each target in a multiplex ELISA-like assay. This study used a Bio-Plex device to read and analyze data.

### 2.4 Statistical analysis

The Chao and Shannon index and cytokines values were compared using a two-way repeated measures analysis of variance (ANOVA) considering the treatment (control and probiotic) and sampling time as variables using the GraphPad Prism Software (37). The PERMANOVA test was applied, considering the same variables to investigate compositional differences in community membership and structure with subsequent pairwise comparisons when appropriate. The analysis of molecular variance (AMOVA) was used for post-hoc comparisons if indicated in the text.

The linear discriminant analysis effect size (LEfSe), which uses a non-parametric factorial Kruskal–Wallis with a subsequent unpaired Wilcoxon test, was applied to detect significant differences in relative abundances associated with the use of probiotics and antibiotics (38).

## 3 Results

### 3.1 Microbiota analysis

A total of 4,249,298 reads were obtained from 140 samples, of which 2,987,277 passed all quality filters and were retained. To normalize the number of reads across all samples and decrease the bias of non-uniform sizes, a subsample of 11,015 reads per sample was used for the analysis. In addition to the negative control, four samples were excluded from the analysis due to low reads.

**Figure 1:**
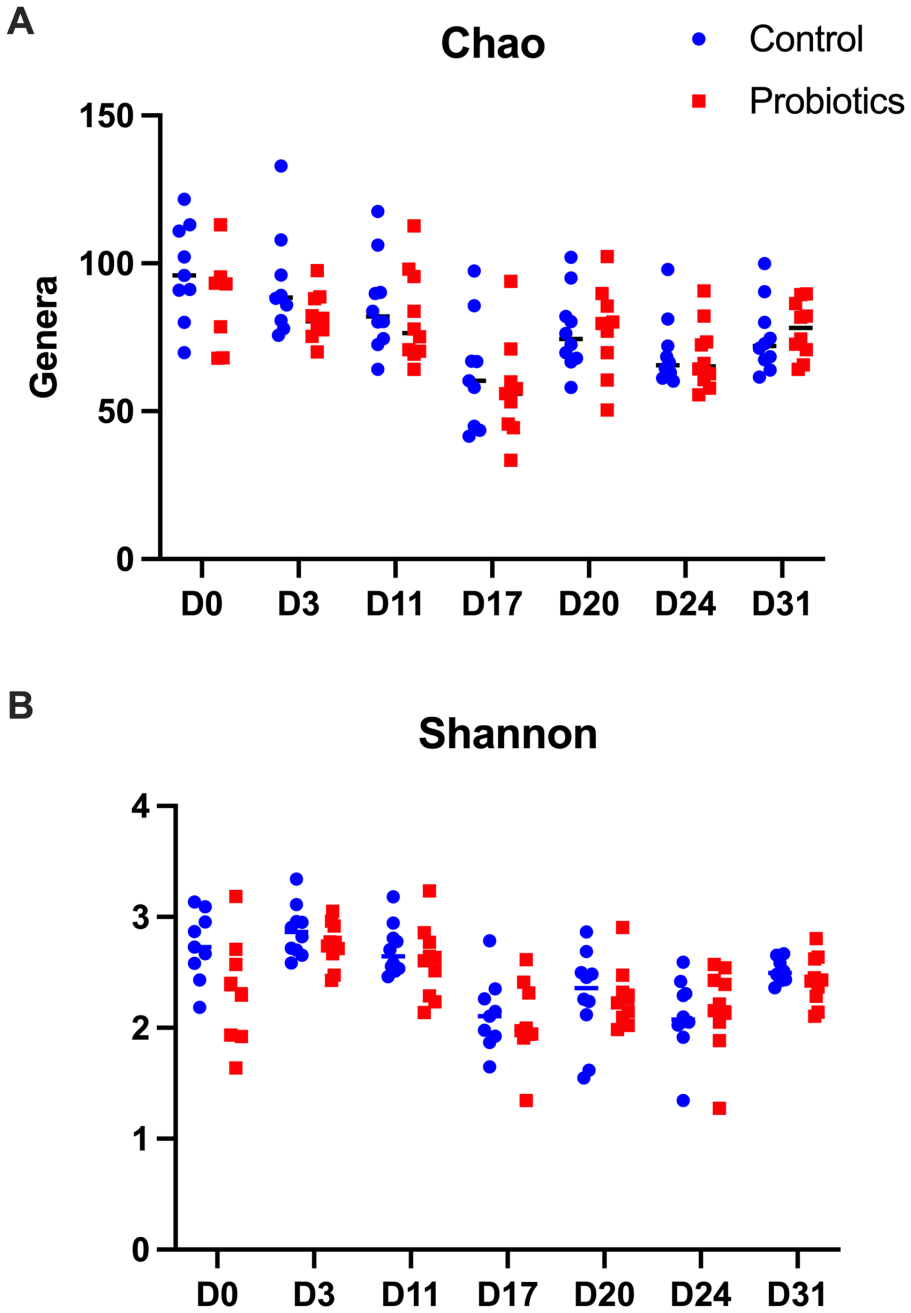
Results of the Chao estimator of richness **(A)** and the Shannon indicator of diversity **(B)** found in 20 dogs at baseline (D0) after 3 days of supplementation with yeast probiotic *Saccharomyces cerevisiae* (D3), before the use of antibiotics (D11), after 5 days of treatment with oral metronidazole (D17) and at days 24 and 31 of the trial. CON, controls; PROB, dogs supplemented with *S. cerevisiae* (1 g/kg PO).

The results of the Chao (richness) and the Shannon (diversity) index are shown in Figure 1. In control dogs, there was a significant decrease in the Chao index after the use of antibiotics (D17) compared to D0, D3 and D11 (*p* = 0.005 and *p* = 0.008, *p* = 0.011, respectively), as well as in D24 compared to D0 (*p* = 0.009). In the PROB group, antibiotics (D17) were associated with lower richness than D0, D11 and D31 (*p* = 0.004, *p* = 0.016, and *p* = 0.016, respectively).

For the Shannon index, samples collected from the CON group on D17, D20 and D24 had a significantly lower diversity than D0 (*p* = 0.018, *p* = 0.024 and *p* = 0.026, respectively). Samples collected on D17, D20, D24 and D31 had lower diversity than samples collected on D3 (*p* < 0.010, *p* = 0.020, *p* = 0.001, and *p* = 0.011, respectively). In addition, there was lower diversity on D17 and D24 compared to D11 (*p* = 0.004 and *p* = 0.030, respectively) but higher diversity on D31 compared to D17 (*p* = 0.032). However, no difference was observed between the control and the probiotic groups.

**Figure 2:**
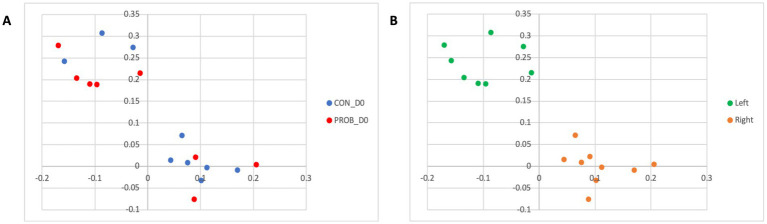
Principal coordinate analysis (PCoA) comparing the similarity of the microbiota membership (Jaccard index) obtained from 20 dogs at baseline (D0) according to the treatment group to which they were assigned **(A)** and according to which side of the room they were housed **(B)**. Samples were collected before the beginning of supplemented with the yeast probiotic *Saccharomyces cerevisiae*. PROB, dogs assigned to the supplementation group; CON, dogs assigned to the control group.

Regarding beta diversity analysis, which considers the taxonomic information, no significant differences between groups were detected on D0 (*p*-value = 0.375). However, two distinct clusters of samples comprised of dogs from both groups (CON and PROB) were found in the membership analysis (Figure 2A), which warned for further investigation. It was found that the samples were clustering according to the side of the room where the dogs were housed: left or right (Figure 2B). A post-hoc analysis comparing dogs housed on the left to those on the right side of the room revealed a significant difference in membership (AMOVA test, *p* < 0.001).

**Figure 3:**
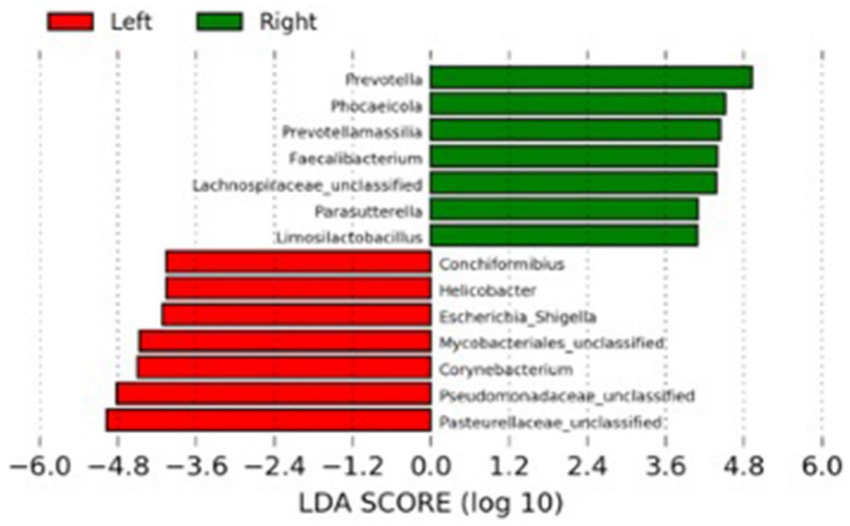
Results of the LEfSe analysis indicating the major bacterial taxa (LDA score >3) statistically more abundant in dogs housed on the left or the right side of the room at the baseline of the study (D0).

The LEfSe analysis was used to characterize the bacterial taxa associated with the different microbiota profiles found on each side of the room at D0. Figure 3 represents differential features with an LDA score >3. Several of the taxa significantly associated with the left side were pathobionts (potentially pathogenic bacteria) generally associated with dysbiosis or inflammation. In contrast, taxa related to dogs on the right side were normal commensals, usually part of a healthy microbiota.

Results of the PERMANOVA test on membership revealed an overall significant impact of treatment with probiotics (*p* = 0.002) and sampling time (*p* = 0.001) but not an interaction between the two variables (*p* = 0.125).

**Figure 4:**
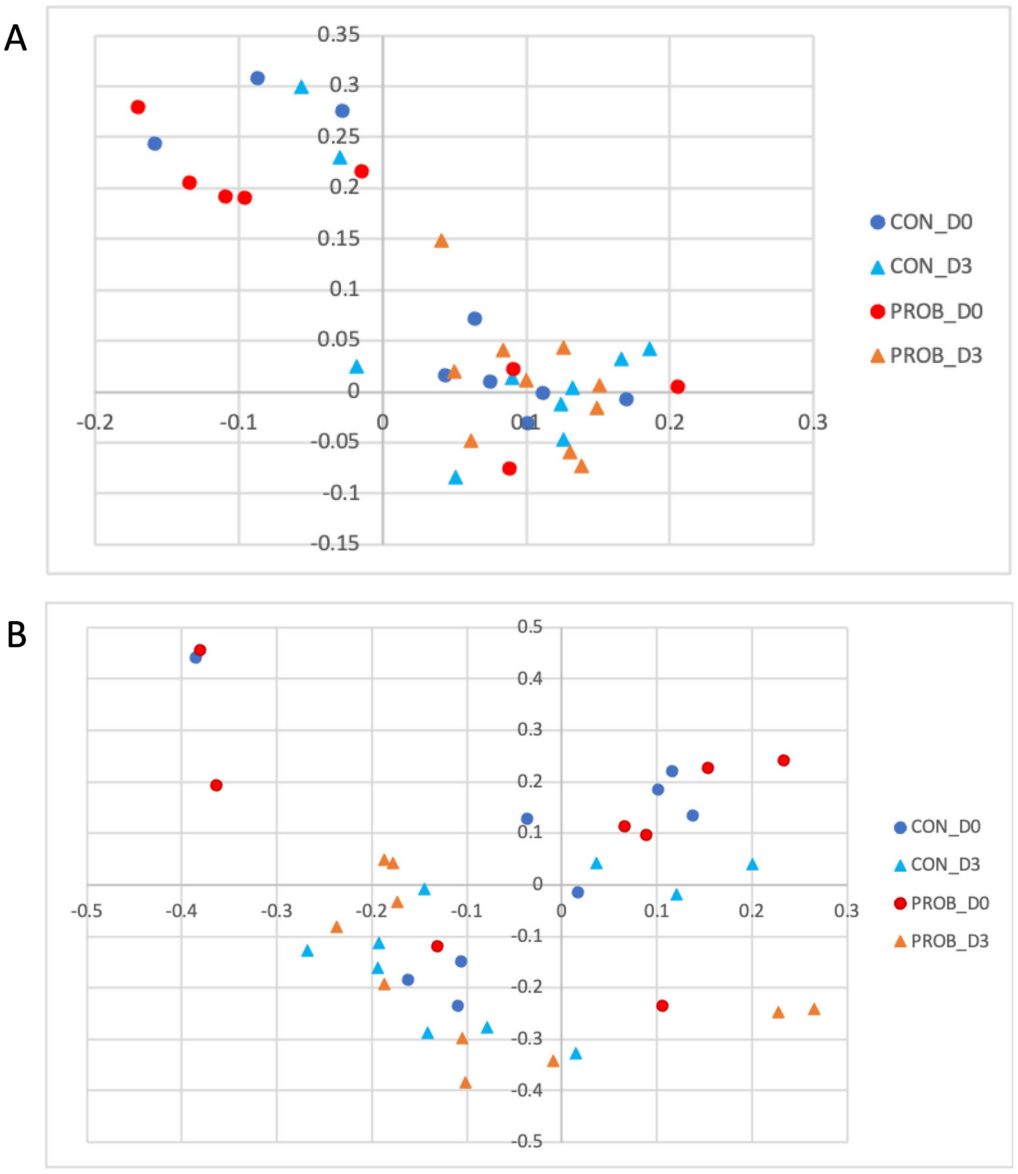
Principal coordinate analysis (PCoA) comparing the similarity of the membership **(A)** and structure **(B)** obtained from controls (CON) and dogs supplemented with *Saccharomyces cerevisiae* (PROB) before (D0) and after 3 days of supplementation (D3).

It can be observed that all five dogs in the PROB group that had an inflammation profile on D0 changed their microbiota composition towards a more healthy microbiota after 3 days of supplementation (Figure 4A). The microbiota structure observed in D0 and D3 is presented in Figure 4B. A posthoc analysis (AMOVA) confirmed that the microbiota structure changed significantly from D0 to D3 in the probiotic group (*p*-value <0.001) but not in the control group (*p*-value = 0.212).

**Figure 5:**
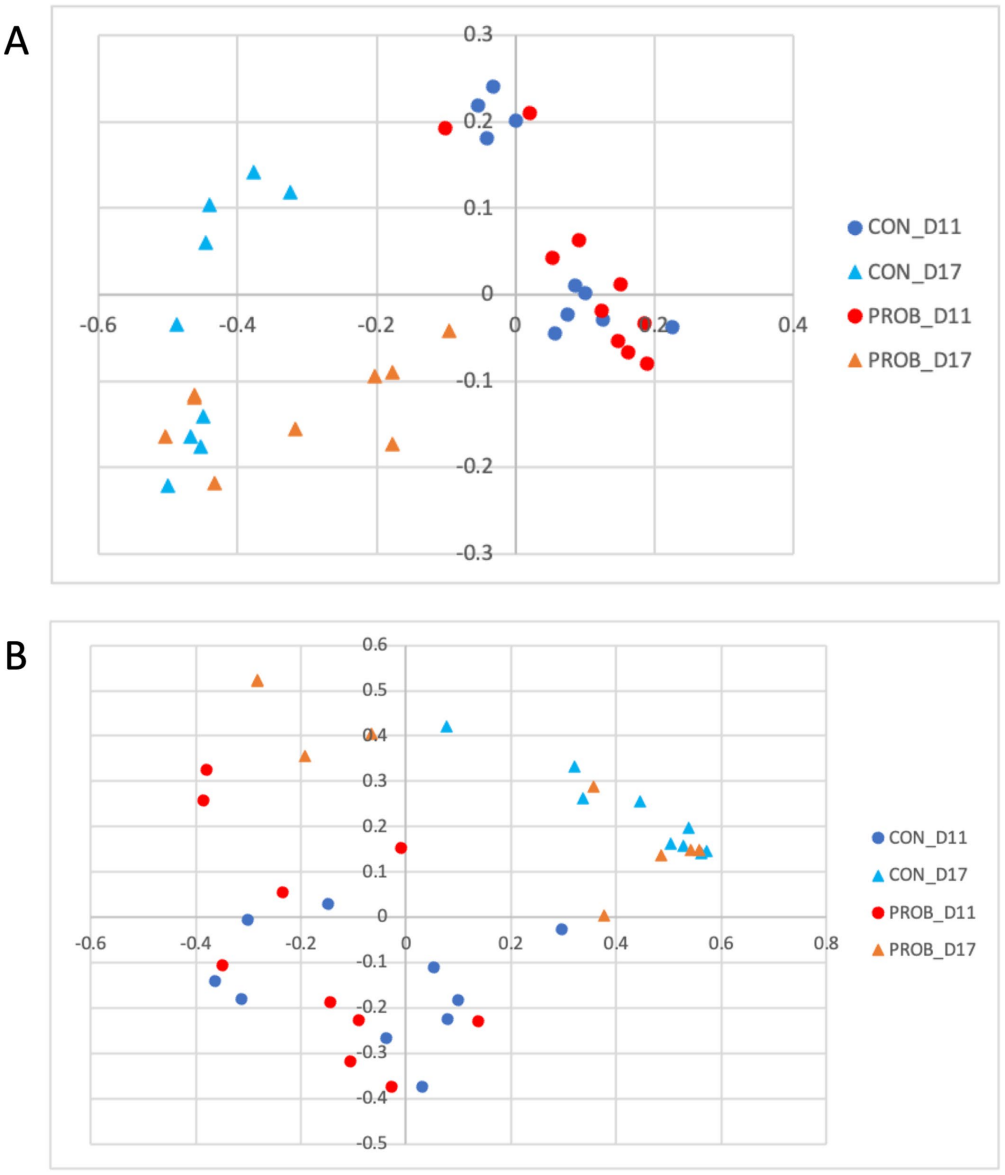
Principal coordinate analysis (PCoA) comparing the similarity of the membership **(A)** and structure **(B)** obtained from controls (CON) and dogs supplemented with *Saccharomyces cerevisiae* (PROB) before (D11) and after (D17) treatment with oral metronidazole for 5 days.

The microbial structure and membership of both groups significantly changed (AMOVA, *p*-value <0.001) after antibiotic treatment (D17) (Figure 5).

**Figure 6:**
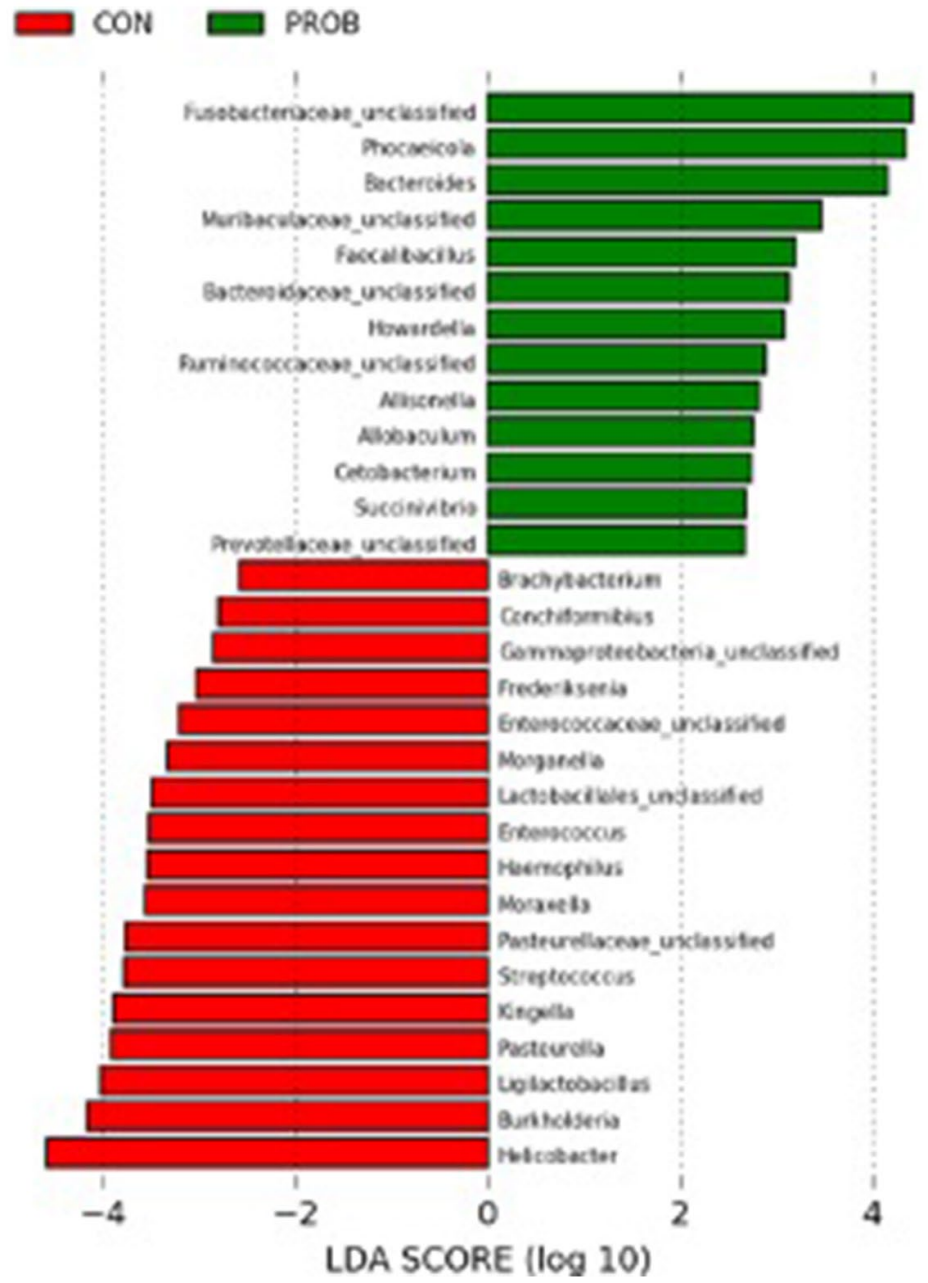
Results of the LEfSe analysis indicating the fecal bacteria significantly different (LDA >2) between controls and dogs supplemented with *Saccharomyces cerevisiae* after treatment with oral metronidazole for 5 days (D17).

The comparison between controls and probiotic-supplemented dogs revealed a significant difference in membership (AMOVA, *p*-value = 0.012) but not structure (AMOVA, *p*-value = 0.234) on D17. Nevertheless, some bacterial taxa significantly differed between CON and PROB during antibiotic-associated dysbiosis on D17, addressed by the LEfSe analysis (Figure 6).

**Figure 7:**
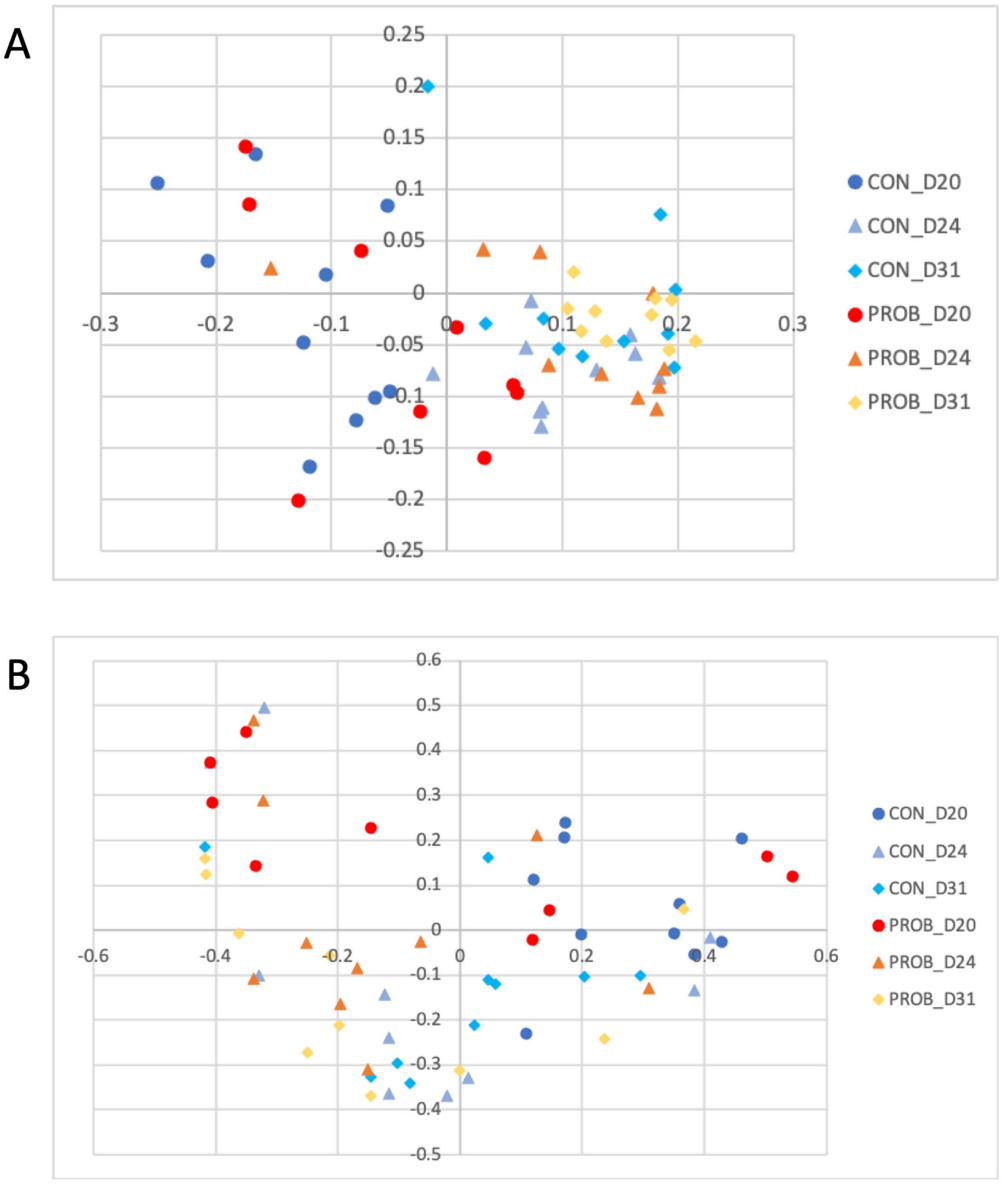
Principal coordinate analysis (PCoA) comparing the similarity of the membership **(A)** and structure **(B)** obtained from controls (CON) and dogs supplemented with *Saccharomyces cerevisiae* (PROB) on days 20, 24 and 31 of the study, investigating the long term impact of yeast supplementation.

There was no difference in either membership or structure between D11 and D24 or D31 (all *p*-values >0.05), suggesting a recovery of the microbiota to its pre-antibiotic state (Figure 7).

### 3.2 Cytokines and cortisol

**Figure 8:**
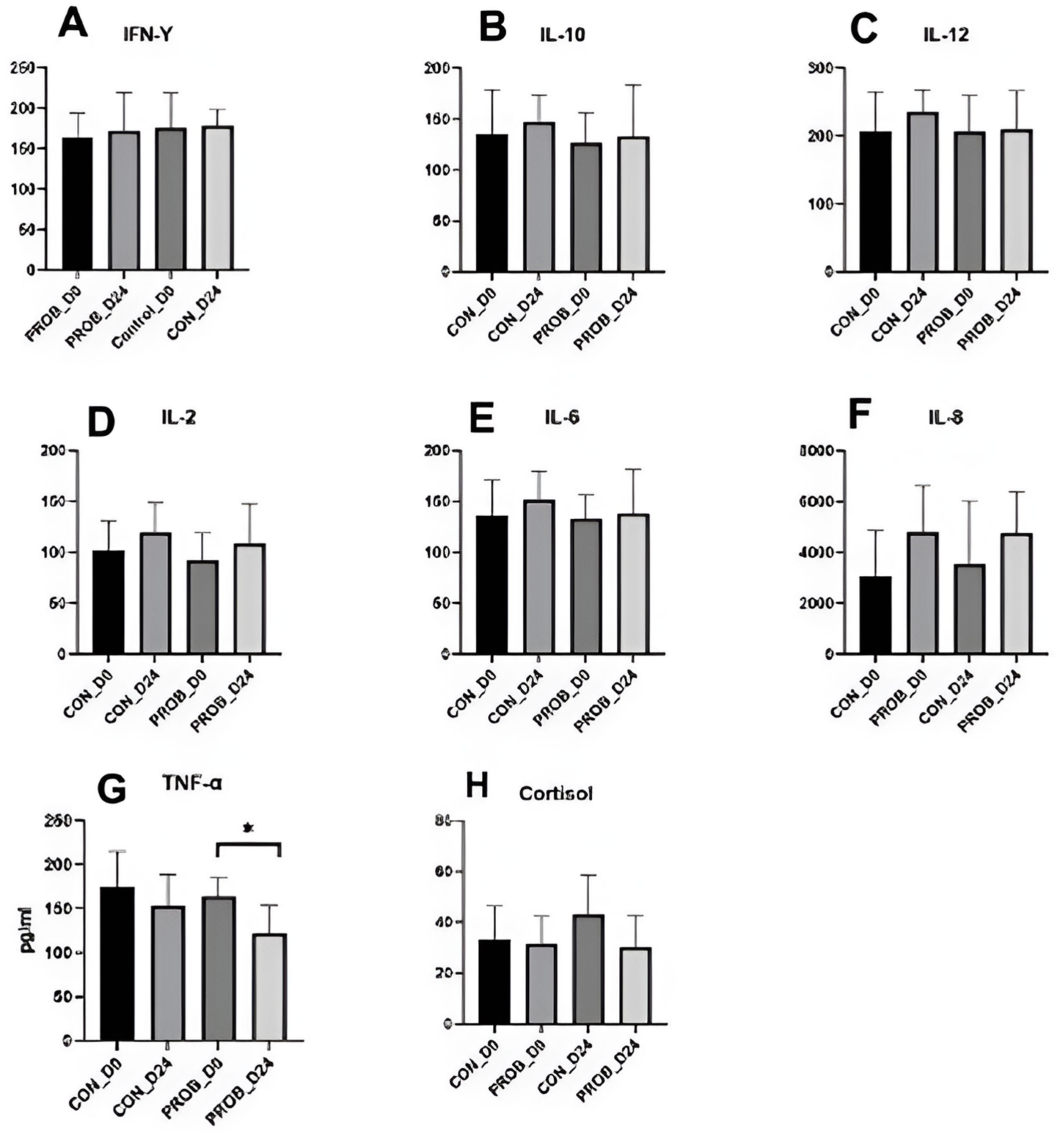
Cytokines **(A–F)** and cortisol levels **(G)** in the serum of 20 dogs supplemented with yeast probiotic Saccharomyces cerevisiae (PROB) and a control group (CON) before (D0) and 24 days after yeast supplementation (D24). ^*^Indicates a significant difference between the time points.

TNF-α levels significantly decreased in the probiotic group from D0 to D24 (*p*-value = 0.03). Still, there was no significant difference in the levels of other cytokines between groups, neither between D0 and D24 (all *p* > 0.05) (Figure 8). In addition, there was no detectable difference in serum cortisol levels.

## 4 Discussion

The objectives of this study were to evaluate the impact of oral administration of the yeast probiotic *Saccharomyces cerevisiae* on the fecal microbiota of healthy adult dogs and to evaluate *S. cerevisiae*’s potential in preventing dysbiosis induced by antibiotics.

Unexpectedly, the microbial composition of dogs at the beginning of the study (D0) formed two distinct clusters according to the side of the facility in which they were housed. Most bacteria associated with dogs on the left side were inflammation-related species, such as *Escherichia*, *Helicobacter*, *Mycobacteria* and Pseudomonadaceae. Conversely, the bacteria with significantly higher abundances in dogs housed on the right side of the room were beneficial species such as *Prevotella*, *Faecalibacterium*, and Lachnospiraceae (39). The reasons that could explain such a marked difference are unknown, but interestingly, dogs from the right side were fed first, while the other dogs were continuously barking. Stress has been shown to influence the physiology of the host intestinal cells with consequences in the bacteria present at the mucosal surface and gut lumen (40). Stressor factors such as transportation can also affect the composition and abundance of the fecal microbiota of dogs (41). They could alter the intestinal motility, further contributing to the differences observed at the baseline of the present study. However, there was no difference in cortisol levels when comparing dogs housed at the different sides of the facility. Therefore, further studies employing more specific stress markers or investigating other factors influencing microbiota composition are necessary to explain this finding. Fortunately, this variable was considered during the designing of the study by evenly distributing dogs from both treatment groups (controls and probiotics) across each side of the room. Noteworthy, the teaching dogs have been fed twice daily to minimize the potential stress related to fasting.

Although the altered baseline microbiota composition might have influenced the impact of probiotics over time, after 3 days of supplementation, all five dogs carrying a stress-related profile reverted to a healthier profile. A previous study using a similar supplementation protocol in Beagle dogs showed an increase in the digestibility of fiber that could be associated with increased energy availability in the form of volatile fatty acids, which are a valuable energy source for enterocytes (i.e., butyrate) and have the capacity to modulate the intestinal microbiota composition positively.

As expected, the microbial composition in the control and the probiotic groups changed drastically after 5 days of treatment with metronidazole. Metronidazole can decrease diversity, cause severe changes in the intestinal microbiota of dogs with diarrhea, and induce dysbiosis in healthy dogs (16, 42). In the present study, supplementation with yeast probiotic *S. cerevisiae* was associated with less severe changes in the fecal microbiota of dogs, as animals receiving the probiotic had statistically different microbiota compared with controls on D17. The LEfSe analysis on D17 revealed that several species associated with the use of probiotics were representative of a healthy canine microbiota (i.e., Fusobacteriaceae, *Bacteroides* spp., *Faecalibacillus* spp., Bacterioidaceae, and Ruminococcaceae).

At the end of the study period (D24 and D31), the microbiota of dogs from both groups had recovered from the dysbiosis caused by metronidazole, as the microbial composition was similar to the normal microbiota before antibiotics (D11).

Antimicrobial drugs are typically used to treat gastrointestinal diseases and are known to cause dysbiosis. However, intestinal microbiota dysbiosis has been associated with GIT disorders such as IBD, food allergies, and infections (43–46). The consequences of the disruption of the intestinal microbiota of dogs remain mainly unknown. Still, beneficial bacteria support the digestion of complex nutrients, synthesize essential vitamins, and produce short-chain fatty acids and might even be involved in behavioural aspects (47). A balanced microbiota protects the gut against harmful pathogens through competitive exclusion and modulation of the immune response and is essential to support optimal mucus production and overall gastrointestinal health (3, 48). Thus, dysbiosis might be a causing or predisposing factor associated with diseases such as chronic enteropathies (49, 50).

In this study, antimicrobial treatment did not cause an increase in inflammation markers 7 days after the end of treatment. Still, TNF-α, often increased during gastrointestinal diseases, statistically decreased from D0 to D24 in dogs supplemented with yeast probiotic *S. cerevisiae*. This was possibly caused by the compositional changes from a stress-related microbiota profile of treated dogs from the left side of the room towards a normal microbiota. However, the low number of dogs used in this study precludes significant conclusions, and larger cohorts enrolling stressed or sick dogs are necessary to investigate this supplement’s anti-inflammatory properties further. Nevertheless, this study analyzed the microbiota of 140 fecal samples of dogs, bringing novel information related to the bacterial dynamics upon the use of yeast probiotics and antibiotic-related dysbiosis. Further studies, including the analysis of intestinal metabolites such as bile acids and glucans, are necessary to reveal the mechanisms by which yeast supplementation benefits the intestinal microbiota.

In conclusion, it was observed that the use of yeast probiotic *S. cerevisiae* was associated with beneficial changes in the microbial composition of dogs carrying a stress-related profile and had the potential to modulate dysbiosis caused by treatment with oral metronidazole. Further studies are justified to evaluate the benefits of supplementation with yeast probiotic *S. cerevisiae* in dogs with gastrointestinal diseases.

## Data Availability

Raw FASTQ files have been deposited at the NCBI SRA Archive under the accession number PRJNA1211059.
